# Differential misclassification between self-reported status and official HPV vaccination records in Japan: Implications for evaluating vaccine safety and effectiveness

**DOI:** 10.1016/j.pvr.2018.05.002

**Published:** 2018-05-25

**Authors:** Manako Yamaguchi, Masayuki Sekine, Risa Kudo, Sosuke Adachi, Yutaka Ueda, Etsuko Miyagi, Megumi Hara, Sharon J.B. Hanley, Takayuki Enomoto

**Affiliations:** a Department of Obstetrics and Gynecology, Niigata University Graduate School of Medical and Dental Sciences, Niigata, Japan; b Departments of Obstetrics and Gynecology, Osaka University Graduate School of Medicine, Suita, Japan; c Department of Obstetrics and Gynecology, Yokohama City University School of Medicine, Yokohama, Japan; d Department of Preventive Medicine, Faculty of Medicine, Saga University, Saga, Japan; e Department of Women's Health Medicine, Hokkaido University Graduate School of Medicine, Sapporo, Japan

## Abstract

Japan has no national vaccine registry and approximately 1700 municipalities manage the immunization records independently. In June 2013, proactive recommendations for the human papillomavirus (HPV) vaccine were suspended after unconfirmed reports of adverse events following immunization in the media, despite no vaccine safety signal having been raised. Furthermore, studies assessing HPV vaccine safety and effectiveness published post suspension are predominantly based on self-reported information. Our aim was to examine the accuracy of self-reported vaccination status compared with official municipal records. Participants were women aged 20–22 yrs, who were attending for cervical screening in Niigata city. Among the 1230 eligible registrants, vaccine uptake, defined as any dose, was 75.0% and 77.2% according to a self-reported questionnaire and municipal records, respectively. The accuracy rate of self-reported information was as follows: positive predictive value (PPV) was 87.7%; negative predictive value (NPV) was 54.5%; sensitivity was 85.2%; and specificity was 59.8%. The validity of self-reported information was only moderate (Kappa statistic = 0.44, 95% confidence interval 0.37–0.50). This combined with the low NPV may lead to reduced estimation of effectiveness and safety. A more reliable method, such as a national HPV vaccine registry, needs to be established for assessing HPV immunization status in Japan.

## 1 Introduction

Incidence of and mortality from cervical cancer continues to increase in young Japanese women of reproductive age [1], [2]. The human papillomavirus (HPV) vaccine was approved in 2009 in Japan. In 2010, the Ministry of Health, Labor and Welfare (MHLW) initiated an expedited promotion project for HPV vaccination, where national government would cover 50% of the total cost, if local government also paid 50%. As a result, public aid was gradually introduced in each municipality for girls aged 12–16 yrs. From April 2013, the HPV vaccine was included in the national immunization program for girls aged 12–16 yrs. However, sensational, unconfirmed reports of adverse events following immunization (AEFI) were extensively broadcast in the media. Consequently, the MHLW decided to suspend proactive recommendation for HPV vaccinations in June 2013 and this is still ongoing [3]. Due to this suspension, three-dose uptake of the HPV vaccine decreased dramatically, from > 70% in those born between 1994 and 1999 to < 1% in those born in 2000 and later [4], [5].

Japan has three levels of government: national, prefectural, and municipal. The nation is divided into 47 prefectures, and each prefecture consists of numerous municipalities, with approximately 1700 in total. At present, 9 vaccines are included in the Japanese childhood national immunization program (NIP) and immunization records for these vaccines are managed by each individual municipality. The records are considered highly sensitive personal information, so it is difficult to obtain access to them and link the records to other databases. Thus, no national vaccine registry exists in Japan.

Unlike studies from the UK or Australia that have used robust population-based vaccine registries when reporting reductions in HPV infection and precancerous lesions [6], [7], clinical studies investigating vaccine safety or vaccine effectiveness in Japan are predominantly based on self-reported vaccination information and not on municipal records [8], [9], [10], [11]. Self-reported information may be susceptible to recall bias and misclassification of vaccination status by self-reporting may result in reduced estimation of effectiveness and safety [12]. When public confidence in the vaccine is low, it is essential that data on vaccination status is as accurate as possible. Therefore, in this study, we aimed to examine the accuracy of self-reported information compared with municipal records of HPV vaccination status in one large Japanese city.

## 2 Material and methods

### 2.1 Participants and survey measures

Participants were 1375 Japanese women aged 20–22 yrs attending for cervical cancer screening in Niigata city between April 2014 and March 2017. Niigata city is located on the northwest coast of Honshu, the largest island of Japan, and with a population of around 180,000, it is the 16th most populous city in Japan. In Niigata, public funding for the HPV vaccine began in 2010 for girls aged 13–16 yrs as part of the expedited promotion project. Since participants were born between April 1994 and March 1997 (aged 14–16 yrs in 2010), they were eligible for free vaccination. Information about individual vaccination status was obtained through a short self-reported questionnaire and from municipal records at Niigata city public health center.

The questionnaire consisted of four questions: one on vaccination status (vaccinated or unvaccinated), one on vaccine type (bivalent or quadrivalent) and two on sexual history; age at sexual debut, and number of sexual partners. Data on sexual history will be presented in a different paper. The questionnaire was sent and returned by post to women who had registered for the study. From the municipal records, we obtained information on date, number of doses and type (bivalent or quadrivalent) of vaccine administered. We defined at least one dose of the vaccine as “vaccinated”.

### 2.2 Statistical analyses

We classified vaccination status of these 1230 women into the following 4 categories:

(1) Confirmed group 1: At least one dose of the HPV vaccine confirmed by both self-reporting and municipal records.
(2) Confirmed group 2: No vaccination confirmed by both self-reporting and municipal records.
(3) Misclassification group 1: At least one dose of the HPV vaccine reported by self-reporting, but not confirmed in municipal records.
(4) Misclassification group 2: No vaccination reported by self-reporting, but at least one dose of the HPV vaccine confirmed by municipal records.

We used student *t*-test for continuous variables and chi-square test for categorical variables. Additionally, we calculated sensitivity, specificity, positive predictive values (PPV), negative predictive values (NPV) and their corresponding 95% confidence intervals (CI). Finally, we calculated a Kappa coefficient to measure agreement between self-report information and municipal records. The kappa value was categorized as follows: almost perfect 0.81 – 1.00, substantial agreement 0.61 – 0.80, moderate agreement 0.41 – 0.60, fair 0.21 – 0.40, and poor < 0.21.

The study protocol was approved by the institutional review boards of Niigata University Graduate School of Medical and Dental Science. Written informed consent was obtained from all participants.

## 3 Results

Of the women attending for screening, both self-reported and municipal records on HPV vaccine status were available for 1230 (89.8%) women. Of these, 145 women were excluded from the analysis due to unavailability of municipal records (n = 5) or no response to the questionnaire (n = 140). For the latter, municipal records showed that 111 women had been vaccinated and 29 women were unvaccinated (data not shown).

Table 1 shows municipal government recorded HPV vaccination status compared to self-reports. While confirmed vaccine coverage was 77.2% (949/1230), self-reported uptake was 75.0% (922/1230). Of those who were vaccinated, 140 (11.4%) had no recollection of actually being vaccinated. This constituted 45.5% (140/308) of respondents who claimed they had not been vaccinated in the self-administered questionnaire. Similarly, 113 (9.2%) of participants claimed they had been vaccinated, when municipal records showed they had not. Thus, in total, 253 (20.6%) of women in this study incorrectly reported their HPV vaccination status. Furthermore, among the 809 women in confirmed group 1, 381 (47.1%), 19 (2.3%) and 409 (50.6%) women answered vaccine type as "bivalent", “quadrivalent” and “unknown", respectively. All 381 women who answered "bivalent" were correct, however, among the 19 women who answered ”quadrivalent”, 57.9% (11/19) had been given the bivalent vaccine (data not shown).

**Table 1:** Actual and self-reported vaccination status (n = 1230).

		Municipal personal records	
		Vaccinated n = 949	Unvaccinated n = 281
Self-reported information	Vaccinated n = 922	Confirmed n = 809 (65.8%)	Misclassification n = 113 (9.2%)
	Unvaccinated n = 308	Misclassification n = 140 (11.4%)	Confirmed n = 168 (13.7%)

Agreement statistics and 95% CIs are shown in Table 2. The sensitivity, specificity, positive predictive value (PPV), and negative predictive value (NPV) were 85.2%, 59.8%, 87.7% and 54.5% respectively. The Kappa coefficient was 0.44 (95% CI: 0.37–0.50) signifying only fair to moderate agreement.

**Table 2:** Agreement statistics and 95% confidence intervals.

Vaccine	HPV	(95% CI^a^)
Confirmed coverage	77.2%	
Self-reported coverage	75.0%	
Kappa	0.44	(0.37–0.50)
Sensitivity	85.2%	(82.8–87.4)
Specificity	59.8%	(53.8–65.6)
Positive predictive value	87.7%	(85.5–89.8)
Negative predictive value	54.5%	(48.8–60.2)

a Confidence Interval.

Information on age at enrollment and vaccine history based on municipal records confirming vaccination is shown in Table 3. Age at enrollment, age at first dose and type of vaccine (bivalent vs quadrivalent) administered were not significant predictors of being able to recall vaccination status or not. The number of doses received, was however, significant. (P = 0.01).

**Table 3:** Personal information on HPV vaccination based on municipal records.

	Vaccination status confirmed n = 949		
	Recollection of vaccination n = 809	No recollection of vaccination n = 140	p value
Age at registration (mean ± SD)	20.4 ( ± 0.7)	20.4( ± 0.8)	0.56
Age at first immunization (mean ± SD)	15.2 ( ± 0.9)	15.2 ( ± 0.9)	0.91
Number of doses, n (%)			0.01
One	6 (0.7%)	5 (3.6%)	
Two	28 (3.5%)	8 (5.7%)	
Three	775 (95.8%)	127 (90.7%)	
Vaccine type, n (%)			0.18
Bivalent	795 (98.3%)	135 (96.4%)	
Quadrivalent	14 (1.7%)	5 (3.6%)	

## 4 Discussion

When public confidence on a vaccine is low, it is essential that data on vaccination status is as accurate as possible. In this paper, we examined the accuracy of self-reported information compared with municipal records on HPV vaccination status in one large Japanese city.

In studies investigating HPV vaccination status and cervical disease outcomes, incorrect reporting of vaccination status may result in differential misclassification, leading to over or under estimation of vaccine effectiveness and/or safety. In the case of vaccine effectiveness, countries such as the UK and Australia use robust population-based registries to report population-level impact of HPV vaccination with regards to reductions in HPV infection, cytological abnormalities and precancerous lesions [6], [7], [13]. Furthermore, one Finnish study which also used population-based registries has reported the first statistically significant decrease in invasive cervical cancer in those vaccinated against HPV [14]. Data from population-based registries is more reliable since it is not subject to recall bias and covers almost all the target population.

Japan has neither a nationwide vaccine registry nor a cervical cytology registry since both vaccination and screening take place at the municipal level [15]. Recently, three studies from Japan reported statistically significant decreases in cervical abnormalities and/or pre-cancerous lesions caused by HPV types 16 and 18 in vaccinated cohorts compared to non-vaccinated cohorts. Ozawa et al. and Tanaka et al. reported that in HPV vaccinated Japanese cohorts who had reached screening age, an 88.1% and 52.1% decrease in ASC-US cytology was observed in HPV vaccinated women aged 20–24 yrs living in Akita and Miyagi prefecture, respectively, compared to those not vaccinated [8], [9]. Cytology results were obtained from prefecture-wide screening data; however, HPV vaccination history was based on a self-reported questionnaire. In both these studies, rates of high grade squamous intraepithelial lesion (HISL) or worse were lower in the vaccinated group, but the decrease was not statistically significant. The reasons for this may have a small sample size in this age-group and not being at an age when high-grade lesions develop. However, misclassification of vaccination status cannot be ruled out. Self-reported vaccination rates in participants in the 1994–1996 birth-cohorts were 42.3–56.9%, respectively. These uptake rates are considerably lower than our results and other reports based on municipal records [4], [5], where uptake was consisently found to be > 70% in this age-group. In the present study, 11.4% of women in this age group had no recollection of vaccination.

Matsumoto et al. reported that HPV16/18 positive rates in cervical intraepithelial neoplasia grade2–3 (CIN2–3) and adenocarcinoma in situ (AIS) were significantly decreased in girls eligible for free HPV vaccination (1994–1995 birth cohorts) compared to those who were not (1986–1993 birth cohorts) [10]. This study is a nationwide multi-center hospital-based study that aims to monitor the population-level impact of the Japanese HPV vaccination program [16]. They reported that the HPV16/18 positive rates in CIN2–3/AIS cases were lower among the vaccinated birth-cohorts (30.0% [6/20]) compared with the unvaccinated cohorts (52.8% [275/521]). However, the difference did not reach statistical significance (p = 0.06). In their study vaccination records are not based on municipal records for the 1994–1995 birth cohorts. Furthermore, the 1986–1993 birth cohorts would have had to self-pay if they received the vaccine and because of this there are no official vaccination records in Japan. Due to the small number of CIN2–3/AIS cases, misclassification of vaccination status may have underestimated vaccine effectiveness.

Even though reports of AEFI with the HPV vaccine continue to appear in the Japanese media, 20.6% of participants in our study misclassified their actual vaccination status. Under-reporting of vaccination status will lead to a smaller denominator and inflated prevalence of AEFI. The potential for differential misclassification to lead to false associations between vaccination and adverse events is concerning. In 2014, in Columbia, for example, in the small town of El Carmen de Bolívar, many girls began to report mobility disorders and fainting after HPV vaccination [17]. As with Japan, this led to a dramatic decrease in HPV vaccine uptake [18]. However, when the Institute of Health carried out a thorough investigation of the symptoms, considering claims that lead poisoning or even the use of Ouija boards had caused the symptoms, they found out that 20% of girls claiming AEFI with the HPV vaccine had not even been vaccinated [19].

Japan also has similar issues with objectively ascertaining HPV vaccination status in relation to safety. When the Japanese Vaccine Adverse Reactions Review Committee (VARRC) decided to suspend proactive recommendations for the HPV vaccine without accurate epidemiological survey, their decision was based not only on reports from health care professionals and/or the vaccine manufacturers, but also on reports from the national HPV vaccine ‘Victims Support Group’. For the latter, it was often the case that there was no information on the type of vaccine used, the number of doses given or the date vaccination took place, making it almost impossible to objectively verify vaccination status [20]. Furthermore, the most recent government initiated epidemiological study to investigate the reported AEFI associated with the HPV vaccine found that 40.3 girls per 100,000 aged 12–18 yrs experienced wide-ranging symptoms like those reported after HPV vaccination, including widespread pain and neurological symptoms, regardless of vaccination status. However, when investigating whether these girls were vaccinated or not, the situation became much more complicated. In this study, vaccination status was based on medical records including self-reports, not on municipal records. For girls who had not been vaccinated the reported rate of the aforementioned symptoms was 20.4 per 100,000, but for girls who had not been vaccinated or their status was unknown (due to no response), it was 46.2 per 100,000. On the other hand, for girls who had been vaccinated, the rate was 27.8 per 100,000 [11]. However, because there is no national HPV vaccine registry in Japan, it is almost impossible to ascertain the actual vaccination status of those who did not respond, making it very difficult to reach any objective conclusions based on the data obtained.

Discrepancy between self-reported information and official vaccination status has also been reported in other countries. One study in the US compared the accuracy of self-reported vaccinated status of 8 different vaccines with electronic medical records. In that study the kappa coefficient of HPV vaccine was 0.67, considerably higher than in the present study. Sensitivity, specificity PPV, and NPV, were 91%, 76%, 80%, and 93%, respectively [21]. While some of these values are similar to the present study, the NPV in our study was much lower than that of US study. The NPV is a measure of ‘false negative’, in other words the extent, in this case, to which those who reported they were not vaccinated were really not vaccinated. Therefore, the NPV depends on the unvaccinated rate. HPV vaccination coverage in the US study and our study was 57.9% and more than 70%, respectively. The main reason for the difference in NPV in both these studies is because of the difference in vaccination coverage. However, it should be noted that 11.4% of our subjects had no recollection of being vaccinated, despite the continual negative news regarding the HPV vaccine in the Japanese media.

This lack of recollection suggests there may have been a lack of education about the HPV vaccine and cervical cancer when the girls were being given the vaccine so that they did not fully understand what vaccine they had been given and why. This may also have serious consequences for future participation in cervical screening, which is low at between 30% and 40%, and even lower in women in their twenties [18], [22]. Studies from both the UK and Australia, where vaccination is given in schools and information about the vaccine along with consent forms is sent from the schools to the parents, have shown that women who were vaccinated against HPV were also more likely to participate in cervical screening [23], [24].

When vaccine confidence is high, misclassification of vaccination status may be a concern if coverage is over-estimated and the desired herd immunity is not achieved. Measles for example needs around 95% uptake to achieve herd immunity [25]. If coverage falls below this, outbreaks may occur. However, when vaccine confidence is low, as with the HPV vaccine in Japan, not being able to objectively verify vaccination status makes it difficult to reassure the public that the vaccine is both safe and effective. This is clearly reflected in the fact that uptake of the HPV vaccine has dropped from > 70% when the vaccine was first publicly funded to < 1% after proactive recommendations for the vaccine were suspended.

Countries like the UK and Australia have childhood vaccination registries making it easier to ascertain actual coverage and investigate reported safety signals by looking at both ecological and linkage data to see if there is any population based increase in certain diseases or conditions before and after the HPV vaccine was introduced, or whether a difference exists in the incidence of these condition in vaccinated and non-vaccinated girls (and boys) [26], [27], [28]. In January 2017, the American Society of Clinical Oncology ASCO) also stressed the importance of implementing nationwide surveillance systems in their Guidelines for the Primary Prevention of Cervical Cancer [29]. In Japan, the MHLW had no risk management nor any risk communication plan. Consequently, instead of being reassured by the government, parents in Japan are receiving mixed messages of having a vaccine that the government doesn’t actively recommend, but still includes in their NIP at no cost for girls of the target age. The government also remains silent regarding the sensational reports of AEFI in the media, which were recently reported by Bonanni et al. to be ‘one of the greatest enemies to HPV vaccination programs’ [30].

This study also has several limitations that must be addressed. The main limitation of our analysis is that we couldn’t obtain information on vaccination when it had taken place in another prefecture or another municipality within Niigata prefecture, or if it had been carried out privately before the introduction of public funding. Since we only had confirmed municipal immunization records from Niigata city, women who were vaccinated in another city or privately would have been classified as “unvaccinated”. Because of this the PPV may have been higher and the NPV may have been lower than reported here.

A second limitation is recall bias because the time interval from vaccination to screening was 4–6 years. This may have been confounded with the fact that those women born between in April 2nd, 1995 to April 1st, 2007, would have also been offered vaccination against Japanese encephalitis between the ages of 14 and 16 years since this vaccine is part of the NIP. Influenza vaccination is also strongly recommended in Japan for school-aged children, but it is not included in childhood NIP, and vaccination coverage is 25–50% in early teens [31]. Both vaccines are recommended as a one dose seasonal vaccine. Only the HPV vaccine is recommended as a 3-dose vaccine over 6 months. Those girls who only received one dose of the HPV vaccine many have confused it with the Japanese encephalitis or flu vaccine and this may explain why correct recollection of whether they had received the vaccine was significantly related to the number of doses administered (Table 3).

A final limitation is that our results may not reflect the general population of Japan, since women who participated in our study were those women undergoing cervical cancer screening and participation in cervical cancer screening in this age group is around 20%. It may be that they had a higher awareness of cervical cancer and how to prevent it and were consequently more likely to have been vaccinated than women of the same age in the general population. Despite this, uptake rates in our study are almost similar to those reported by Hanley et al. in Hokkaido where municipal records were also used [4].

A publicly funded HPV vaccination began in 2010 in Niigata, the sixteenth most populous city in Japan. We were able to access personal vaccination records managed by the municipal government, only after strict approval from our institution's ethics review. There are approximately 1700 municipalities in Japan and each one manages vaccination history of its residents, making it almost impossible to obtain Japan wide data. Furthermore, women (or men) who were not vaccinated using public funding will not be included in any official records. This not only makes it more difficult to assess vaccine effectiveness and safety but also during the past four years, girls who were eligible to receive the HPV vaccine with public funding, but chose not to due to suspension of proactive recommendations, may want to be vaccinated when proactive recommendation resumes. In fact, it has been suggested that the MHLW should include these girls in a catch-up vaccination program [32]. However, most of them will be outside the target age group for free vaccination, and many may have gone to university or started working in a city other than the one where they were eligible to receive free vaccination. Without a proper national HPV vaccine registry, it may be almost impossible to ascertain who is eligible for the catch up and who isn’t. Finally, should future clinical data show that a booster vaccination is necessary, it will also be impossible to identify who should receive this booster.

## 5 Conclusions

The validity of self-reported information in the present study was only moderate (Kappa statistic = 0.44, 95% confidence interval 0.37–0.50) This, combined with the low NPV, may mean that in other areas of Japan which rely on self-reporting for HPV vaccination status, reduced estimation of effectiveness and safety may or may have already occurred, since we have demonstrated that considerable misclassification does exists between self-reported HPV vaccination status and official municipal records. To be able to convince Japanese citizens, as well as the politicians controlling whether proactive recommendations for the HPV vaccine should be resumed, that the HPV vaccine is both safe and effective, it is essential to have robust objective data on vaccination status. Therefore, the government needs to establish a more reliable method, such as a national HPV vaccine registry, for assessing HPV immunization status in Japan.
